# A Lesson from America

**Published:** 1921-06-18

**Authors:** 


### A Lesson from America.

Following our recent appeal to the Ministry of Health for a greater propagandist effort on systematic and modern lines, a newspaper correspondent draws attention once more to the up-to-date methods adopted in the United States, as exemplified in the States Life Extension Institute. The pamphlet which this Department issued some time ago containing a set of rules for healthy living based on modern science and presented in an attractive form is just the kind of useful service which the publicity branch of a Government Department ought to perform. The following points as to the correct carriage of the body are given to indicate the method of approach. A stooping posture or "slouch" leads to a chronic relaxation of the abdominal muscles, which by causing a slight displacement of the organs of the body produces absorption of toxins or poisons which would otherwise pass away. These poisons lower the resistance of the body to disease. When the body, either sitting or walking, is held in an erect posture, such as soldiers are trained to assume, the abdominal muscles remain taut and cause the blood to circulate in a normal, healthy manner, and so forth. A correspondent of the *Express* states that the careful distribution of this one pamphlet is claimed to have brought about a substantial improvement in the health and habits of many Americans. We are convinced that on similar lines our own Ministry of Health can accomplish valuable results. The point to remember is that the British public has gone far beyond the stage when it was happy in its own ignorance and was genuinely suspicious of all attempts at health reform, particularly those which directly concerned the individual person. The British public, speaking generally, is now eager enough to learn how to become and keep healthy. It is an opportunity not to be missed.

